# The mediating effects of dysfunctional attitudes and moderating effect of sex between stressful life events and depressive symptoms among Chinese college students

**DOI:** 10.1038/s41598-023-38103-y

**Published:** 2023-07-05

**Authors:** Wenfu Li, Jingting Chen, Yujia Liu, Yanzhi Liu, Xiaoran Hu, Fuqin Mu, Chuanxin Liu, Ying Zhang, Yan Liu

**Affiliations:** 1 School of Mental Health, Jining Medical University, Jining, 272013 China; 2 Teachers’ Union, Jining Medical University, Jining, 272067 China; 3 Melbourne School of Psychological Sciences, The University of Melbourne, Parkville, VIC 3010 Australia; 4 School of Clinical Medicine, Jining Medical University, Jining, 272013 China; 5 Graduate School of Arts and Sciences, Columbia University, New York, 10027 USA; 6 School of Public Health, University of Sydney, Sydney, NSW 2006 Australia

**Keywords:** Human behaviour, Risk factors

## Abstract

Stressful life events (SLEs) closely correlates with depressive symptoms. Although vulnerability-stress model suggests SLEs interacted with dysfunctional attitudes (DA) to predict depression, the mediation role of DA is poorly understood. Therefore, this study intended to investigate the mediating role of DA and the moderating role of sex between SLEs and self-reported depression. A cross-sectional survey was conducted with a sample of 7769 Chinese college students. Participants were assessed in terms of self-reported SLEs, DA and depression variables. Results showed that there were significant sex differences in both SLE and DA. DA mediated the association between SLE and self-reported depression. The moderated mediation model analysis showed that the interaction of SLEs and sex significantly predicted DA in mediator variable model and self-reported depression in dependent variable model. Results indicated that DA partially mediated the association between SLEs and self-reported depression, and sex moderates the association between SLEs and both DA and self-reported depression, which females have bigger changes of DA and depressive symptoms across low and high levels of SLEs than males.

## Introduction

The depressive symptoms of college students has become a major public health problem^1^. College students are the most vulnerable group to depressive symptoms due to that they were in a specific development phase and needed to accommodate to the new life schedule and built new relationships with others^2,3^. College students had a higher levels of depressive symptoms than that of general population^2,4^ and their matched community peers^5^. The overall prevalence of depressive symptoms among Chinese college students was reported as 28.4% by a meta-analysis of 113 independent articles with 185,787 participants^6^. College students with depressive symptoms might develop low cognitive function and high risk of suicidal ideation or suicidal behaviors^7,8^. Given the negative influence of depressive symptoms, it was necessary to explore the possible factors that contributed to depressive symptoms and identify the underlying mechanisms of these factors in college students.

### Stressful life events and depressive symptoms

Meta-analysis and reviews indicated that the strongest psychological predictive factors of depressive symptoms were high neuroticism, negative repetitive thinking, low self-esteem, self-criticism, perceived injustice, and stressful life events^9–12^. Among these predictive factors, stressful life events experienced recently were particularly crucial to notice. Stressful life events are those events which affect, threaten, or damage the people’s physical and psychological health^13^. Numerous studies found that stressful life events were closely related with depressive symptoms in variety of groups, such as children, adolescents and adults^14–16^. Other studies also indicated that the increment of stressful life events could result in depressive symptoms^17,18^. However, when facing stressful life events, some people developed apparent depressive symptoms while other people remained health and not developed poor psychological outcomes^19^. Some study even concluded that the exposure to early life stress might have a potential protective effects against later stressors^20^. The fact, that not everyone who encounters stressful life events inevitably suffers from depressive symptoms, indicated that the influence of stressful life events on individual psychological development might be affected by other psychological traits^14^. That is, the association between stressful life events and depressive symptoms might be mediated and moderated by other variables.

### Mediating role of dysfunctional attitudes

Dysfunctional attitudes refers to those attitudes and beliefs which generated negative concepts about self, others, and the future^21^. Beck’s cognitive vulnerability-stress model regards dysfunctional attitudes as “My value as a person depends greatly on what others think of me” or “If I fail at work, then I am a failure as a person”, which increase the risk of depressive symptoms after experienced stressful life events^22,23^. Sun et al.^24^ pointed out that dysfunctional attitudes were one of the central characteristic of depression. Empirical studies indicated that dysfunctional attitudes were significantly related to depressive symptoms in child, adolescent and adult samples^25–27^. Beck^22^ proposed that dysfunctional attitudes was a central component of negative schema which was closely associated with the onset and duration of depressive symptoms. Therefore, it is reasonable to assume that dysfunctional attitudes are positively related to depressive symptoms.

In addition, negative life events and childhood trauma could lead to dysfunctional attitudes. Previous studies indicated that stressful life events were correlated with dysfunctional attitudes in adolescents and adults^28^. Flouri and Panourgia^29^ found that life stress was a risk factor for dysfunctional attitudes in 10–19 years old adolescents. Other studies also indicated that childhood adverse events were positively related to dysfunctional attitudes in college students^21^ and patients with depressive disorders^30^. These studies indicated that negative life experiences, such as childhood trauma and stressful life events, were possible antecedents of dysfunctional attitudes. Numerous studies found the interaction effect of life events and dysfunctional attitudes consistently predicted depression^31–34^, however, the mediating effect of dysfunctional attitudes between stressful life events and depressive symptoms has not been explored. Furthermore, some studies revealed that dysfunctional attitudes mediated the relationship between individual factor, environmental factor and depressive symptoms^35–37^. Thus, it is reasonable that the relationship between stressful life events and depressive symptoms might be mediated by dysfunctional attitudes.

### Moderating role of sex

Sex difference is an important factor to investigate the association between stressful life events, dysfunctional attitudes and depressive symptoms. Previous studies explored the sex differences in dysfunctional attitudes of college students and patients with depression, and found mixed results. Some studies found that there were sex difference in dysfunctional attitudes in college students in Turkey, China, and America^38–40^, while other studies found that there were no sex difference^41^. The results of sex difference in dysfunctional attitudes of patients with depression were also conflicted with each other^42,43^. In addition, numerous studies have investigated the sex difference in the reactions to stressful life events, and found that the negative reaction of females were stronger than that of males^44^. In contrast, other studies found there were no significant sex difference in the response to stressful life events^45,46^. Females were more susceptible to negative events than males in family, which in turn caused elevated depressive symptoms^47^.These conflicted results indicated that it was important to consider the moderating effect of sex in considering the negative influence of stressful life events. Nevertheless, few studies directly explored the effect of sex in the association among stressful life events, dysfunctional attitudes and depressive symptoms.

### The present study

**Figure 1:**
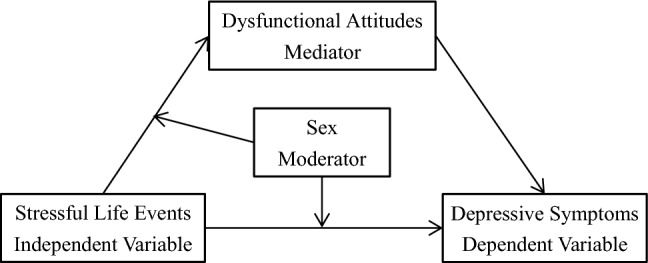
The hypothetical moderated mediation model.

Although previous studies indicated that there were a significant relationship between stressful life events and depressive symptoms^48^, how stressful life events relates to depressive symptoms is less understood. Based on previous studies and theories, the present study came up with four hypotheses and constructed a moderated mediation model (displayed in Fig. 1) to examine the following hypotheses. Hypothesis 1: Stressful life events positively associated with depressive symptoms of Chinese college students. Hypothesis 2: Dysfunctional attitudes mediated the association between stressful life events and self-reported depression. Hypothesis 3: Sex moderated the mediating effect of dysfunctional attitudes. Hypothesis 4: Sex moderated the association between stressful life events and depressive symptoms.

## Methods

### Sampling and participants

Using cluster sampling, we recruited 8079 freshmen from Jining Medical University, including the main campus locating in Jining and the satellite campus locating in Rizhao, and Weifang Medical University locating in Weifang. Jining, Rizhao and Weifang are cities in Shandong province, P.R. China. These participants came from different majors, such as clinical medicine, public health and stomatology, and different provinces of China. The data was collected using Questionnaire Star Platform (www.wjx.cn). Because this platform could be set to answer all items before successful submission, this guaranteed that no missing values in the recovered questionnaires. All participants completed the informed consent before the survey began. The research program was approved by the Research Ethics Committee of Jining Medical University. All methods were carried out in accordance with the relevant guidelines and regulations in China. Among 8079 participants, 310 participants were excluded from further data analysis due to demographic data errors, straight line response, regular response or pattern response. At last, we collected 7769 valid questionnaires with effective recovery rate of 96.16%.

Table 1 shows the demographic characteristics of participants. It showed that, in the sample of 7769, there were 3101 (39.95%) males and 4668 (60.05%) females. The age of participants was 18.37 ± 0.86 years old and ranged from 13 to 30. Majority of the participants came from rural (63.06%) and 2889 (36.94%) participants came from urban. There were 2988 (38.37%) only-child participants and 4781 (61.63%) non-only-child participants. 5527 (71.29%) participants majored in medicine and 2242 (28.71%) participants majored in non-medicine.

**Table 1:** The demographic characteristics of participants (*N* = 7769).

Parameters		*N* (%)
Sex	Male	3101 (39.95%)
	Female	4668 (60.05%)
Residence	Rural	4880 (63.06%)
	Urban	2889 (36.94%)
One child	Only-child	2988 (38.37%)
	Non-only-child	4781 (61.63%)
Major	Medicine	5527 (71.29%)
	Non-medicine	2242 (28.71%)

### Measures

#### Adolescent self-rating life events check-list (ASLEC)

The Chinese version of ASLEC compiled by Liu et al.^49^ was used to assess life events including academic pressure, family, health, punishment, personal relationship, and other events that happened within past 12 months. This scale consisted of 27 self-assessment items. Participants were asked about the feeling of some specific life events with a potential for causing distress, and rated how the event negatively influenced themselves using a five-point Likert scale with five answer options ranging from nothing to severest. The score of ASLEC was equal to the sum of 27 items. The higher the score of ASLEC, the more of stressful life event was. This scale had satisfactory reliability and validity revealed by Liu et al.^49^. The Cronbach’s *α* coefficient was 0.83 in the present research.

#### Dysfunctional attitude scale (DAS)

The Chinese version^39^ of DAS form A^49^ was used to measure the presence and intensity of dysfunctional attitudes. This scale was a self-report scale and consisted of 40 items which was rated on a seven-point Likert-type scale. Each scale had seven forced-choice options ranging from fully disagree to fully agree. There were ten items need reverse coding. The total score equaled to the sum of the numerical response values of the 40-items. The higher the total score, the more dysfunctional were the individual’s style of thinking. We used the Chinese version of the DAS translated by Lin et al.^39^, which has a satisfactory psychological quality. The Cronbach’s *α* coefficient of DAS in present study was 0.91.

#### Beck depression inventory (BDI)

The Chinese version^50^ of BDI-II^51^ was used to assess the severity of depressive symptoms in adults and adolescents populations. This self-reported inventory included 21 self-report items and assessed specific physical, cognitive, and affective performance of depression based on a two-week time period^51^. Each item consisted four representations that vary according to the severity of depression and was rated on a four-point scale ranging from 0 to 3, with a score of “0” representing the absence of symptoms and a score of “3” representing the severe symptoms. The total score was calculated by adding the numerical response values for all 21 items. Higher the total score presented greater severity of depressive symptoms. This Chinese version had satisfied reliability and validity^50^. The Cronbach’s *α* in the present study was 0.91.

### Data analysis

SPSS 22.0 was used to conduct the descriptive statistical analysis. The model 8 in PROCESS 3.3 macro compiled by Hayes^52^ for SPSS was used to test the mediation model and moderated mediation model. In the moderated mediation model, in which sex was regarded as the moderating variable, the interaction effect of stressful life events × sex predicted the dependent variable (depression) and mediating variable (dysfunctional attitudes). Demographic variables including age, residence, one-child and major were included as control variables. All variables were standardized before running the moderated mediation model. The bootstrap method was used to construct the 99% confidence interval with 10,000 resamples. If zero was not included in the 99% confidence interval, the effect was statistically significant.

## Results

### Descriptive statistics

Table 2 showed the descriptive statistics of the study variables.

**Table 2:** The descriptive statistics of the study variables.

	*M* (*SD*)	Range
ASLEC	17.91 (12.75)	0–105
DAS	131.36 (24.04)	40–280
BDI	3.32 (5.61)	0–61

*ASLEC* adolescent self-rating life events check-list, *DAS* dysfunctional attitude scale, *BDI* beck depression inventory.

Table 3 provided the sex difference in study variables. It showed that the score of ASLEC of males was lower than that of females (*t* = − 5.15, *P* < 0.001, Cohen’s *d* = 0.12) and the score of DAS of males was higher than that of females (*t* = 7.21, *P* < 0.001, Cohen’s *d* = 0.17). There was no significant sex differences in the score of BDI.

**Table 3:** Sex difference in study variables.

Variable	Male		Female		*t*	*P*	Cohen’s *d*
	Mean	*SD*	Mean	*SD*			
ASLEC	16.99	13.48	18.51	12.20	− 5.15	< 0.001	0.12
DAS	133.76	26.01	129.76	22.50	7.21	< 0.001	0.17
BDI	3.23	5.96	3.38	5.35	− 1.14	0.255	0.03

*ASLEC* adolescent self-rating life events check-list, *DAS* dysfunctional attitude scale, *BDI* beck depression inventory.

### Correlation analysis

Table 4 showed the results of Pearson correlation analysis of study variables. Results revealed that the score of both ASLEC and DAS was positively correlated with BDI.

**Table 4:** Correlation analysis results.

	ASLEC	DAS	BDI
ASLEC	1		
DAS	0.22***	1	
BDI	0.32***	0.32***	1

*ASLEC* adolescent self-rating life events check-list, *DAS* dysfunctional attitude scale, *BDI* beck depression inventory. ***P < 0.001.

### Dysfunctional attitudes as a mediator

**Figure 2:**
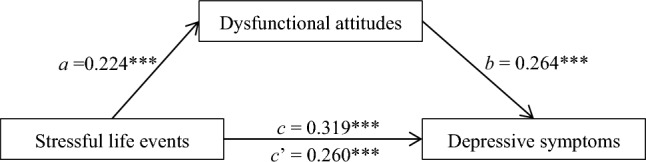
The mediating role of dysfunctional attitudes between stressful life events and self-reported depression. The mediation model was adjusted for age, sex, urban/rural, only-child/non-only-child and major. Path coefficients (a, b, c and c’) were standardized regression coefficients. c total effect of stressful life events on depressive; c’ direct effect of stressful life events on depressive. ***P < 0.001.

Adjusted for age, sex, urban/rural, only-child/non-only-child and major, the mediating effect of dysfunctional attitudes between stressful life events and self-reported depression was tested using PROCESS 3.3. All variables were standardized before running the mediation model. The results were shown in Fig. 2. The total effect (path c) of stressful life events measured by ASLEC on self-reported depression measured by BDI was statistically significant (*β* = 0.319, *P* < 0.001, 99% CI from 0.291 to 0.347). The path a (*β* = 0.224, *P* < 0.001, 99% CI from 0.195 to 0.252) and path b (*β* = 0.264, *P* < 0.001, 99% CI from 0.236 to 0.291) were also significant, which indicated that there were a positive relations between stressful life events and dysfunctional attitudes, and dysfunctional attitudes and self-reported depression. The indirect effect of dysfunctional attitudes between stressful life events and self-reported depression was 0.059 (a × b) and the 99% CI was 0.048 to 0.072, which revealed that the mediating effect of dysfunctional attitudes was statistically significant. The ratio of indirect effect to total effect was 18.75%. Further, the direct effect (path c’) of stressful life events on self-reported depression was also significant (*β* = 0.260, *P* < 0.001, 99% CI from 0.232 to 0.287), which indicated that the association between stressful life events and self-reported depression was partially mediated by dysfunctional attitudes.

### Moderated mediation model analysis

**Figure 3:**
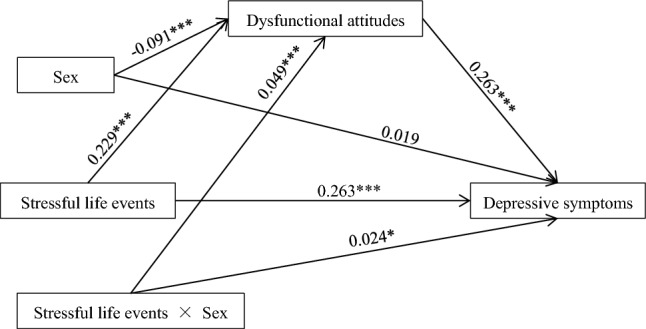
The moderated mediation model between stressful life events and depressive symptoms. *P < 0.05, **P < 0.01, ***P < 0.001.

The SPSS macro PROCESS 3.3 (Model 8) developed by Hayes^52^ was used to test the assumed moderated mediation model that the indirect and direct association between life events and self-reported depression were moderated by sex. The analysis results were displayed in Table 5 and Fig. 3. The interaction of stressful life events and sex significantly predicted dysfunctional attitudes (*β* = 0.049, SE = 0.011, *P* < 0.001) in mediator variable model and self-reported depression (*β* = 0.024, SE = 0.010, *P* = 0.020) in the dependent variable model. Therefore, sex moderated both the association between stressful life events and dysfunctional attitudes, and the association between stressful life events and self-reported depression.

**Table 5:** Testing the moderated mediating effects: sex as the moderator (*N* = 7769).

	Model 1 (DAS)			Model 2 (BDI)		
	*β*	*t*	99% CI	*β*	*t*	99% CI
Age	0.017	1.53	− 0.012, 0.046	0.017	1.61	− 0.010, 0.044
Residence	− 0.039	− 3.14**	− 0.071, − 0.007	0.005	0.41	− 0.025, 0.035
One child	− 0.007	− 0.52	− 0.040, 0.026	0.001	0.11	− 0.030, 0.032
Major	0.026	2.37*	− 0.002, 0.055	− 0.026	− 2.47*	− 0.053, − 0.001
ASLEC	0.229	20.53***	0.200, 0.257	0.263	24.42***	0.235, 0.290
Sex	− 0.091	− 7.94***	− 0.121, − 0.062	− 0.019	1.73	− 0.009, 0.047
ASLEC x Sex	0.049	4.46***	0.021, 0.077	0.024	2.33*	− 0.003, 0.050
DAS				0.263	24.59***	0.235, 0.290
*R*^2^	0.06			0.17		
*F*	70.68***			196.70***		

All *β* values were standardized regression coefficients.

*ASLEC* adolescent self-rating life events check-list, *DAS* dysfunctional attitude scale, *BDI* beck depression inventory.

*P < 0.05, **P < 0.01, ***P < 0.001.

**Figure 4:**
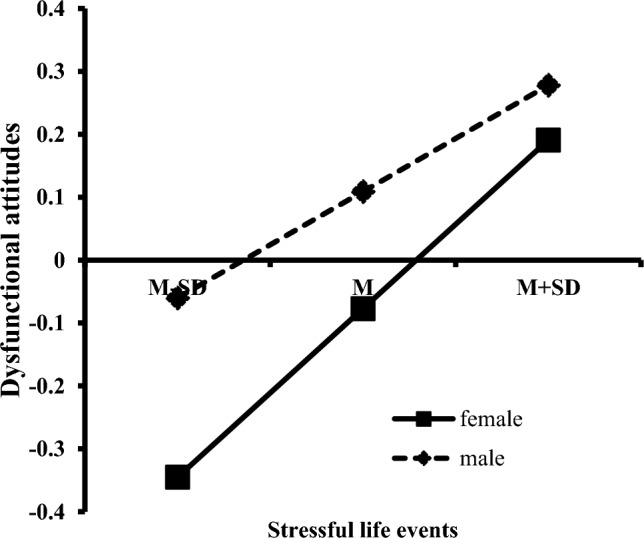
Interaction effect of stressful life events and sex on dysfunctional attitudes.

**Figure 5:**
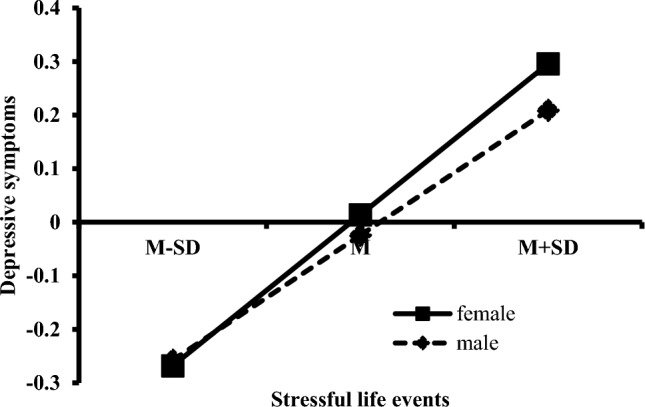
Interaction effect of stressful life events and sex on depressive symptoms.

Further, simple slope analysis was used to test these interaction and investigate whether slopes for the male group were different from that of the female group in model 1 and model 2. The simple slope figures were displayed in Figs. 4 and 5. As shown in Fig. 4, the effect of stressful life events on dysfunctional attitudes was stronger for female students (*β* = 0.268, *t* = 18.02, *P* < 0.001, 99% CI [0.230, 0.306]) than that for male students (*β* = 0.169, *t* = 10.23, *P* < 0.001, 99% CI [0.127, 0.212]). In other words, the dysfunctional attitudes of female students were more likely to be influenced by stressful life events than that of male students. As shown in Fig. 5, the effect of stressful life events on self-reported depression was stronger for female students (*β* = 0.282, *t* = 19.75, *P* < 0.001, 99% CI [0.245, 0.319]) than that for male students (*β* = 0.233, *t* = 14.92, *P* < 0.001, 99% CI [0.193, 0.274]). That is, female students more easily experienced depressive symptoms when inevitably confronted higher stressful life events than male students. These results indicated that stressful life events interacted with sex, such that female students had bigger changes of both dysfunctional attitudes and depressive symptoms across low and high levels of stressful life events than male students. Male students reported higher level of dysfunctional attitudes and lower level of depressive symptoms when they suffered from stressful life events, while female students reported lower level of dysfunctional attitudes and higher level of depressive symptoms.

## Discussion

In the present study, a large (*N* = 7769) sample of Chinese college students with high representativeness was enrolled to investigate the relationship between stressful life events and self-reported depression. The mediating effect of dysfunctional attitudes and the moderating effect of sex were also investigated. Our results showed that stressful life events was positively related to depressive symptoms, and this relationship was mediated by dysfunctional attitudes. Meanwhile, both the direct and indirect pathway of this mediation model were moderated by sex. Female students exhibited a greater relation between stressful life events and depressive symptoms, and stressful life events and dysfunctional attitudes compares to male students. This indicated that females may be more vulnerable to the negative influence of stressful life events on dysfunctional attitudes and depressive symptoms. This results have important contributions to the research on stressful life events, dysfunctional attitudes, and depressive symptoms and have great practical significance for mental heath education workers.

### Sex difference in study variables

Although the focus of our study was not to explore the sex difference in these variables, our results showed that there was significant sex differences both in stressful life events and dysfunctional attitudes. Previous study consistently found that the scores of stressful life events of female college students were higher than that of male college students^53^. Contrary to the findings of Jiang et al.^54^, male adolescents experienced more stressful life events than female adolescents. In addition, consistent with the findings of Gotlib^55^, males had significantly higher DAS scores than females. Haeffel et al.^56^ also found that male college students scored higher on the DAS than female students. However, the sex difference in dysfunctional attitudes were not consistently exhibited^42,43^. You et al.^53^ suggested that the conflicting sex difference in dysfunctional attitudes might resulted from the intricate mutual relation of dysfunctional attitudes with other diathesis of cognitive theory of depression. Additionally, previous studies across different countries found that depressive symptoms occurred more than twice as frequently in female adults than in male adults^57,58^, but the present study did not found significant sex difference in self-reported depression. Hankin et al.^59^indicated that measures, such as BDI only assessed a shorter time period, cannot uncover sex difference in depressive symptoms. The discrepant sex difference in stressful life events, dysfunctional attitudes, and depressive symptoms suggested that there might be other factors mediated or moderated the association between stressful life events, dysfunctional attitudes and depressive symptoms. Further systematic studies need to reveal the nature of relationships between pathogenic factor and depressive symptoms.

### The effects of stressful life events on depressive symptoms

Our results indicated that stressful life events were more easy to result in depressive symptoms, which was in correspondence with previous research^9^. Numerous studies recently indicated that stressful life events was associated with self-reported depression in children, adolescents and adults^14–16^. Other studies also proved stressful life events could more easily lead to depressive symptoms^17,18^. Given the consistency of the results indicated the association between stressful life events and depressive symptoms, both college and family should reduce the frequency of stressful life events to promote the mental health of college students.

### The mediating role of dysfunctional attitudes

This study found that dysfunctional attitudes mediated the association between stressful life events and self-reported depression. Dysfunctional attitudes was consistently correlated with depressive symptoms, anxiety and stress^60^. Longitudinal study also indicated that dysfunctional attitudes was an important predictor of depressive symptoms^61^. The cognitive theory of depression pointed that dysfunctional attitudes might make individuals collapse to get depressed when experienced negative life events^33^. The diathesis-stress model of depression suggested that the dysfunctional attitudes might play as diathesis or predisposing factor, which could lead to the feeling of depression when individuals come through stressful life events^62^. Previous studies found that dysfunctional attitudes moderated the association between stressful life events and depressive symptoms^31–33^. That is, individuals with high score in dysfunctional attitudes might be more easily experienced depressive symptoms when confronted stressful life events than others with low score in dysfunctional attitudes. Other longitudinal researches also indicated that the interaction effect between stressful life events and dysfunctional attitudes was positively predicted depressive symptoms^63,64^. Our result further indicated that dysfunctional attitudes mediated the connection between stressful life event and self-reported depression in Chinese college students. That is, stressful life events influenced depressive symptoms through the mediating effect of dysfunctional attitudes. More researches were needed to investigate the mediating role of dysfunctional attitudes between stressful life events and depressive symptoms in other samples, such as patients with major depressive disorder or healthy adolescents.

### The moderating role of sex

Moreover, the current results showed a significant moderating role of sex on the connection between stressful life events and both dysfunctional attitudes and self-reported depression. Further simple slope analysis indicated that these connections were stronger for females than males. This results were consistent with previous researches, which explored the different reactions of each sex to stressful life events and found that female students tend to respond more strongly than male students^44^. One population-based study found that there was an interaction between negative life events and sex, as the risk rate of major depression was significantly higher in females than in males, particularly in individuals with low stress exposure^65^. Thus, the present results indicated that female college students were more susceptible to stressful life events. However, Jordanova et al.^66^ found that sex did not moderate the connection between stressful life events and common mental disorders in a large sample of adult. Given that study focused on common mental disorders, measured by Clinical Interview Schedule compiling to assess psychiatric disorder^67^, as an outcome, our results might not be directly comparable.

### Limitations

There were some drawbacks in our study. In the first place, this study used a cross-sectional method which displayed not a causation but a correlation between study variables. Further longitudinal studies are needed to validate these findings. In the second place, all the data of this study were based on self-reported questionnaires which involved numerous actual individual experiences and inner feelings. The present results might be influenced by the effects of social prejudice and self-protection. Future studies could examine these variables through classmates estimate, parents estimate, or experimental study. In the third place, the samples enrolled in the present study were only from two medical universities in Shandong Provence which might influence the external validity of conclusions. Subsequent researches need to investigate more college students from more universities in China. In the end. This study only investigated the mediating effect of dysfunctional attitudes and moderating role of sex between stressful life events and self-reported depression, but did not examine the influence of other variables, such as perceived social support, coping style, and self-efficacy, which were all closely associated with depressive symptoms^68–70^. Future researches could investigate the effects of these variables between stressful life events and depressive symptoms.

## Conclusions

In summary, the current results further validated the influence of stressful life events on self-reported depression among a larger sample of Chinese college students. The results illustrated the mediating role of dysfunctional attitudes in the pathway from stressful life events to depressive symptoms. In addition, we also found evidence of two-way interaction, indicating that sex moderated the association between stressful life events and depressive symptoms, and stressful life events and dysfunctional attitudes. Female students had bigger changes of both dysfunctional attitudes and depressive symptoms across low and high levels of stressful life events than male students.

## Data Availability

The data is available on request to the corresponding author.
